# A traffic light enzyme: acetate binding reversibly switches chlorite dismutase from a red- to a green-colored heme protein

**DOI:** 10.1007/s00775-020-01784-1

**Published:** 2020-04-03

**Authors:** Durga Mahor, Julia Püschmann, Menno van den Haak, Pepijn J. Kooij, David L. J. van den Ouden, Marc J. F. Strampraad, Batoul Srour, Peter-Leon Hagedoorn

**Affiliations:** 1 Department of Biotechnology, Delft University of Technology, Van der Maasweg 9, 2629HZ Delft, The Netherlands; 2 Present Address: Institute for Integrative Biology of the Cell (I2BC), CEA, CNRS, Univ. Paris‐Sud, Université Paris‐Saclay, 91198 Gif‐sur‐Yvette Cedex, France

**Keywords:** Chlorite dismutase, Green heme protein, Electron paramagnetic resonance (EPR), Acetate binding, Charge transfer band

## Abstract

**Abstract:**

Chlorite dismutase is a unique heme enzyme that catalyzes the conversion of chlorite to chloride and molecular oxygen. The enzyme is highly specific for chlorite but has been known to bind several anionic and neutral ligands to the heme iron. In a pH study, the enzyme changed color from red to green in acetate buffer pH 5.0. The cause of this color change was uncovered using UV–visible and EPR spectroscopy. Chlorite dismutase in the presence of acetate showed a change of the UV–visible spectrum: a redshift and hyperchromicity of the Soret band from 391 to 404 nm and a blueshift of the charge transfer band CT1 from 647 to 626 nm. Equilibrium binding titrations with acetate resulted in a dissociation constant of circa 20 mM at pH 5.0 and 5.8. EPR spectroscopy showed that the acetate bound form of the enzyme remained high spin *S* = 5/2, however with an apparent change of the rhombicity and line broadening of the spectrum. Mutagenesis of the proximal arginine Arg183 to alanine resulted in the loss of the ability to bind acetate. Acetate was discovered as a novel ligand to chlorite dismutase, with evidence of direct binding to the heme iron. The green color is caused by a blueshift of the CT1 band that is characteristic of the high spin ferric state of the enzyme. Any weak field ligand that binds directly to the heme center may show the red to green color change, as was indeed the case for fluoride.

**Graphic abstract:**

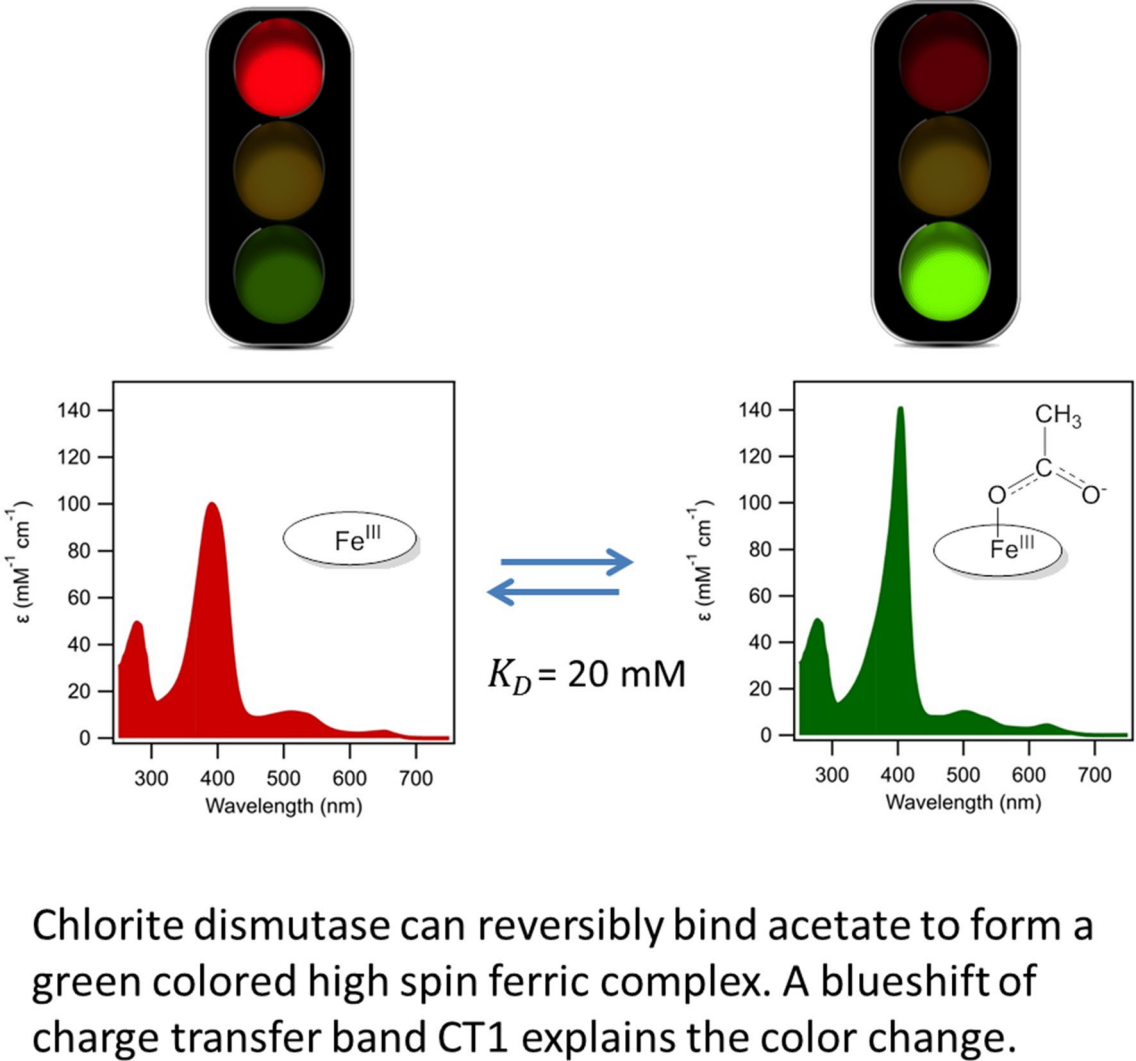

**Electronic supplementary material:**

The online version of this article (10.1007/s00775-020-01784-1) contains supplementary material, which is available to authorized users.

## Introduction

Chlorite dismutase is an essential enzyme in (per)chlorate-respiring bacteria [1, 2]. The enzyme catalyzes the disproportionation of the toxic compound chlorite ClO_2_^−^, the endproduct of (per)chlorate respiration, into harmless Cl^−^ and O_2_. This heme b containing enzyme is unique as it catalyzes O=O bond formation which is otherwise only catalyzed by the oxygen-evolving manganese cluster of photosystem II [3]. Chlorite dismutase has been extensively studied structurally and spectroscopically [4–7]. However, to date, the precise mechanism of the enzyme remains elusive.

Genomic studies have revealed two distinct classes of chlorite dismutase (Cld), penta- and hexameric Cld or dimeric Cld [8]. Furthermore, phylogenetic and structural relationships exist with the coproheme decarboxylase HemQ and the dye decolorizing peroxidases DyP [9, 10]. HemQ is involved in heme biosynthesis in bacteria that belong to the Firmicutes and Actinobacteria. DyPs are enzymes with a broad substrate scope and are unrelated to other types of peroxidases. Together all these protein clades form the peroxidase-chlorite dismutase superfamily [11].

**Fig. 1:**
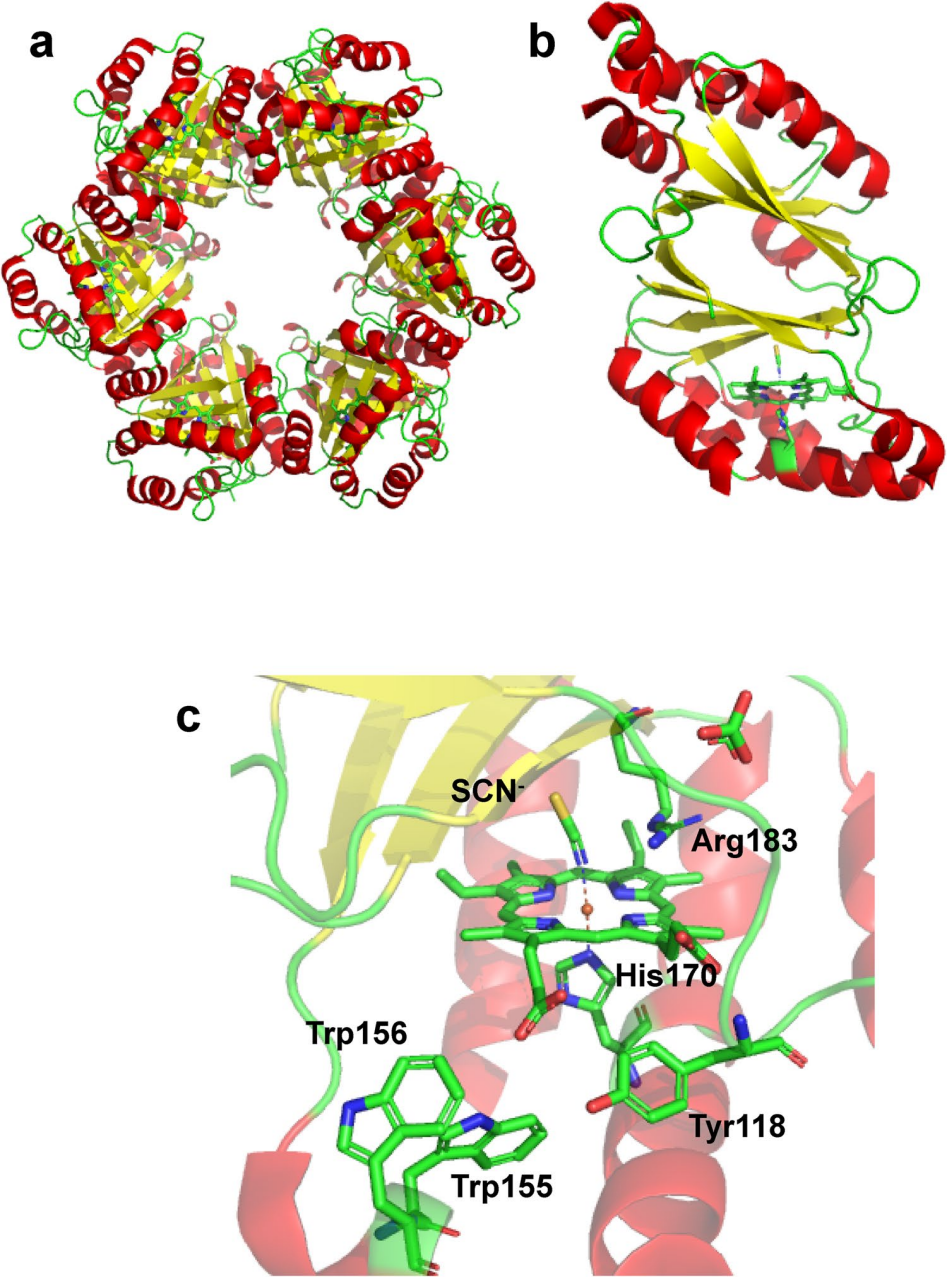
Crystal structure of *Ao*Cld (PDB 2vxh). **a** The crystal structure discloses the hexameric form of *Ao*Cld [6]. The heme cofactor and important residues are depicted as sticks. **b** The monomeric form of *Ao*Cld is showing the secondary structure elements. **c** The active site architecture of *Ao*Cld with bound thiocyanate at the active site. Figures were generated using PyMOL [49]

The dimeric Cld enzymes are structurally (subunit structure and interface, conformational as well as thermal stability) and spectroscopically different. Dimeric Clds are from bacteria that are not chlorate-respiring but can catalyze chlorite conversion. The biological function of these enzymes is not clear, although the organisms contain nitrate reductases that may reduce chlorate non-productively to chlorite [12, 13]. Trp156 close to the heme is highly conserved in pentameric Cld, but not in dimeric Cld. The crystal structure of recombinantly expressed chlorite dismutase from *Azospira oryzae* (*Ao*Cld) shows that it is a homohexamer (although likely a pentamer in solution), in which each monomer consists of two domains with a ferredoxin-like fold. The active site comprises a heme b cofactor with an axial His170 and a proximal Arg183 (Fig. 1) [6]. The conserved Arg183 adopts a so-called “in” position, where the guanidinium group is pointing towards the heme group, or “out” position, where the guanidinium group is pointing towards the entry of the substrate channel.

The UV–visible spectrum of *Ao*Cld is characteristic of a penta-coordinated high spin (HS) ferric heme protein. The Soret peak is broad and has a peak maximum at 391 nm. The Q bands are broad featured around 510 nm and the charge transfer CT1 band is found at 647 nm. The sixth coordination position is available to the substrate and different ligands. Cld has been found to bind (predominantly anionic) ligands: fluoride, cyanide, azide, thiocyanate, nitrite and imidazole [4, 6, 14, 15]. The R183A mutation has been found to abolish azide and fluoride binding, but not imidazole in *Da*Cld, which suggests that Arg183 stabilizes anionic ligand binding by hydrogen bond donation [16].

The activity and spectroscopic properties of Cld are highly pH-dependent [4, 16, 17]. Upon examination of the pH dependence of the activity and stability of *Ao*Cld we discovered that the enzyme solution became green-colored in acetate buffer pH 5.0 (Fig. S1). The color change was reversible after buffer exchange due to the reversible binding of acetate as a ligand to the heme iron in *Ao*Cld. To our knowledge, the binding of acetate directly to the heme iron is almost unique to Cld. The UV–visible and EPR spectroscopic properties of the green-colored acetate bound *Ao*Cld are reported and compared to the fluoride and imidazole adducts of *Ao*Cld.

## Materials and methods

### Chemicals

Antibiotics (kanamycin and chloramphenicol), hemin and IPTG procured from Sigma. Buffer components and purification reagents were purchased from Sigma and Merck.

### Protein production and purification

The strains and plasmids that were used are given in Table S1. *Ao*Cld was recombinantly expressed in *E. coli* using the pET28a-*Ao*Cld construct in *E. coli* BL21(DE3) pLysS as described elsewhere [18]. The expression strain was cultivated in TB medium containing 50 µg mL^−1^ kanamycin, 25 μg mL^−1^ chloramphenicol and 60 μg mL^−1^ hemin (pre-dissolved in 1.4 N NaOH) in a 15 L fermenter (Applikon) at 37 °C. The fermenter was stirred at 750 rpm with an air inflow of 5 L min^−1^. Expression was induced by the addition of 119 μg mL^−1^ IPTG when the culture reached an OD_600_ = 0.5. The cultivation temperature was reduced from 37 to 25 °C after induction. The cells were harvested by centrifugation 7 h after induction using a Sorvall centrifuge at 17,000*g* for 10 min at 4 °C. The cell pellet was washed twice using 20 mM Tris–HCl pH 7.5 containing 500 mM NaCl and 50 mM imidazole (Buffer A). The resulting *Ao*Cld containing cells were resuspended in Buffer A containing additionally 1 mM PMSF, 1 mM MgSO_4_, 200 mg L^−1^ lysozyme and 10 μg mL^−1^ DNase. The cell suspension was homogenized with a Homogenizer RW16 (IKA) and subsequently disrupted using a cell disruptor (Constant Systems) operating at 1.5 kbar. The cell-free extract (CFE) was obtained as the supernatant after centrifugation using a Sorvall centrifuge at 17,000*g* for 90 min at 4 °C. The CFE was filter sterilized using a 0.22 μm Steritop filter (Millipore) before protein purification.

*Ao*Cld was purified from the CFE using IMAC chromatography as the recombinant protein contained an N-terminal His_6_ tag. A 150 mL Ni Sepharose 6 Fast Flow column (GE Healthcare) was equilibrated with 4 column volumes (CV) buffer A using an NGC medium pressure liquid chromatography system (Bio-Rad) at a flow rate of 3 mL min^−1^. 1.5 L CFE was loaded onto the column using a flow rate of 0.7 mL min^−1^ overnight, and subsequently, the column was washed using 1.5 CV buffer A at a flow rate of 3 mL min^−1^. *Ao*Cld was eluted from the column using a linear gradient from 0–100% 20 mM Tris–HCl pH 7.5 containing 150 mM NaCl and 500 mM imidazole (buffer B) in 4 CV and 2 CV 100% buffer B. *Ao*Cld started to elute at circa 325 mM imidazole.

*Ao*Cld was subsequently desalted to remove excess imidazole with HiTrap Desalting columns (GE Healthcare) using the NGC system (Bio-Rad) with 20 mM Tris–HCl pH 7.5 (flow rate 4 mL min^−1^). The buffer was exchanged to 50 mM KPi pH 7.0 using a gravitation flow desalting column PD10 (GE Healthcare) according to the manufacturer’s instructions. To further remove bound imidazole the protein was extensively dialyzed against 100 mM KPi pH 7.0 to a dilution factor of 650 with a dialysis cycle of a minimum 8 h using a dialysis membrane with a 3.5 kDa cutoff and a diameter of 16 mm (Visking dialysis tubing, Medicell Membranes) at 5 °C. The purified *Ao*Cld was concentrated to 8 mg mL^−1^ using a centrifugal ultrafiltration device with a 30 kDa cutoff (Amicon filter, Millipore) and stored at − 80 °C until further use. The concentration of pure *Ao*Cld was determined spectrophotometrically using *ε*_280_ = 52.41 mM^−1^ cm^−1^ (monomer concentration).

*Ao*Cld R183A was produced with the QuikChange kit (Agilent) using the mutagenic primers *Ao*Cld-R183A-F 5′-CTGGTGAATGTCAAAGCGAAGTTGTATCAC-3′ and *Ao*Cld-R183A-R 5′-GTGATACAACTTCGCTTTGACATTCACCAG -3′ with the mutagenic codon underlined. *E. coli* pET28a *Ao*Cld-R183A was cultivated in 1 L TB medium as described above. The enzyme was purified using the same procedure as described above for the WT enzyme.

### Biochemical characterization

The protein concentration was determined according to the Bicinchoninic acid method using the BCA protein assay kit (Uptima, Interchim) according to the manufacturer’s instructions [19]. The protein purity was examined using SDS-PAGE using the Criterion XT gel system (Bio-Rad). UV–visible spectra of *Ao*Cld were measured using the Cary 60 spectrophotometer (Agilent) at 21 °C. *Ao*Cld activity was measured polarographically as oxygen is produced during the reaction using the Clark-type oxygen electrode (type 5331, YSI Life Science) in 100 mM KPi pH 7.0 using 1 mM sodium chlorite as substrate at 20 °C.

### Equilibrium binding titrations

Equilibrium binding titrations were performed using acetate, fluoride, and imidazole as ligands by monitoring the change of the Soret band. The UV–visible spectrum of *Ao*Cld was monitored and 0–180 mM sodium acetate was titrated to 10 µM *Ao*Cld in 100 mM citrate–phosphate buffer pH 5.0 and 100 mM KPi pH 5.8. For acetate as a ligand, the absorbance difference between the Soret maximum at 404 nm and the isosbestic point at 396 nm was plotted against the unbound ligand concentration. For the titration with fluoride as a ligand 0–120 mM NaF was titrated against 10 μM *Ao*Cld in 100 mM KPi pH 7.0 and the absorbance difference between the Soret maximum at 400 nm and the isosbestic point at 390 nm was plotted. For imidazole 0–1 mM ligand was titrated against 10 µM *Ao*Cld in 100 mM KPi pH 5.0, 100 mM KPi pH 5.8, 100 mM KPi pH 7.0 and 100 mM Tris buffer pH 8.0, and the absorbance difference between 412 nm and the isosbestic point at 404 nm was plotted. The data were fitted to Eq. 1.

1 $$\mathrm{A}\mathrm{B}\mathrm{S}={\mathrm{A}\mathrm{B}\mathrm{S}}_{0}-{\mathrm{A}\mathrm{B}\mathrm{S}}_{\mathrm{m}\mathrm{a}\mathrm{x}}\frac{[L]}{{K}_{D}+[L]}$$

*K*_*D*_ is the dissociation constant. [*L*] represents the unbound ligand concentration, which was assumed equal to the total ligand concentration for acetate and fluoride. In the case of imidazole, the unbound ligand concentration was corrected for the bound ligand concentration using the experimentally determined extent of saturation and the total enzyme concentration.

### EPR spectroscopy

EPR measurements were performed using a Bruker EMXplus 9.5 spectrometer and the following conditions: 9.402 GHz microwave frequency, 0.2 mW microwave power, 100 kHz modulation frequency, 10 Gauss modulation amplitude and a temperature of 20 K, unless stated otherwise. The low temperature was maintained by boiling liquid helium and cold helium vapor was passed through a double-wall quartz glass tube, which was mounted and fitted in the rectangular cavity [20, 21]. Samples were prepared containing 75 μM (monomer concentration) *Ao*Cld in 100 mM KPi pH 7.0 containing ligands or 100 mM acetate buffer pH 5.0 to afford a final volume of 200 µL.

## Results

### Bound imidazole was difficult to remove during *Ao*Cld purification

Overexpressed *Ao*Cld was purified through a Nickel Sepharose column with elution buffer of 20 mM Tris–HCl and 150 mM NaCl, 500 mM imidazole pH 7.5. The high imidazole concentration in the elution buffer causes the occupancy of the vacant site of the heme iron in *Ao*Cld. The UV–visible spectrum of the Nickel Sepharose purified *Ao*Cld showed a sharp Soret peak at 413 nm with a high absorptivity as well as α and β bands at 562 and 535 nm, which indicates a 6-coordinated low spin (LS) ferric heme of the imidazole complex (Fig. S2a). Desalting (HiTrap Desalting and PD10) steps removed bound imidazole and the Soret peak has blue-shifted with a decreased absorptivity at 406 nm. Although a significant fraction of the bound imidazole was removed by the desalting, still an imidazole bound *Ao*Cld fraction remained. EPR spectroscopy showed that only 30% of the imidazole was removed at this stage, as determined by the characteristic LS signal of the imidazole adduct (*g*_*z*,*y*,*x*_ = 2.96, 2.25, 1.51). This is consistent with the high affinity of *Ao*Cld for imidazole, for which a *K*_*D*_ = 8 μM has been reported [4].

To further clean the enzyme, *Ao*Cld was dialyzed extensively and the Soret band blue-shifted from 406 to 391 nm, which is comparable to the Soret previously reported for native *Ao*Cld [4] (Figure S2a). During the whole process of purification, the *R*_z_ ratio A_391nm_/A_278nm_ improved after each step (Table S2) [22]. *Ao*Cld was purified with homogeneity of > 90% as inferred from SDS-PAGE. SDS-PAGE analysis revealed the single band presence of 30 kDa (Fig S2b).

### A color change of chlorite dismutase upon ligand binding

**Fig. 2:**
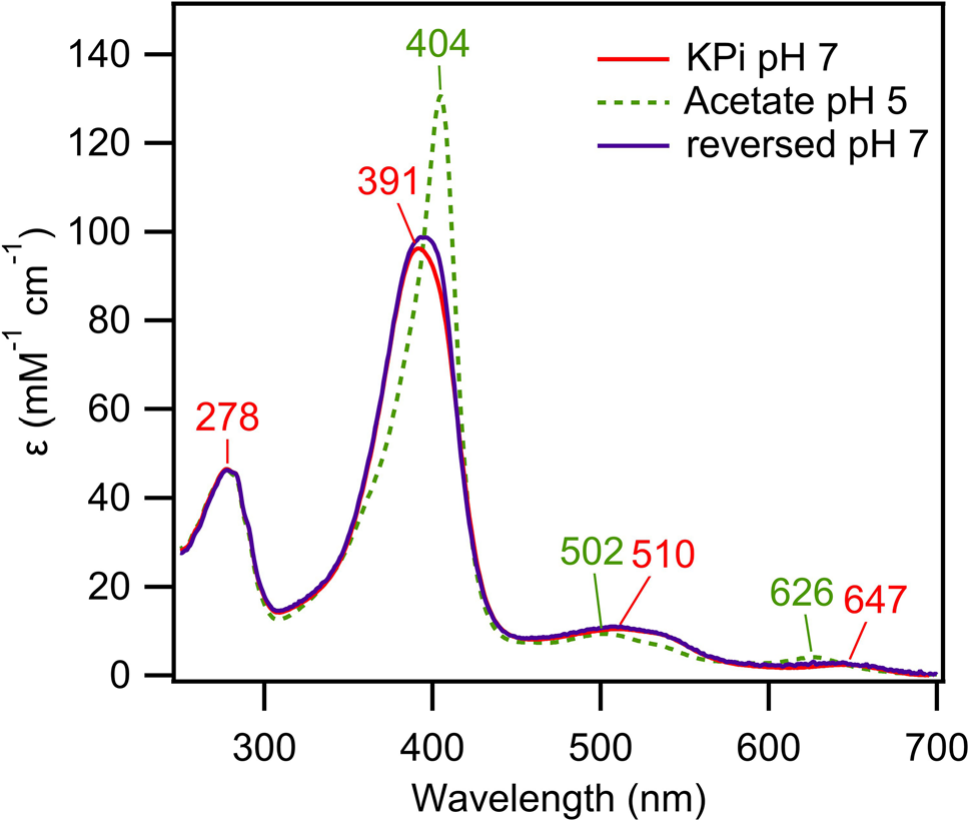
Reversibility of acetate binding to *Ao*Cld. UV–visible spectra of 10 µM *Ao*Cld solution in 100 mM KPi pH 7.0 before buffer exchange (red line), after buffer exchange to acetate buffer pH 5.0 (green dotted line) and after subsequent buffer exchange to KPi pH 7.0 (purple line)

It was observed that *Ao*Cld switches its characteristic red color to green after buffer exchange from 100 mM KPi pH 7.0 to 100 mM sodium acetate buffer pH 5.0 (Fig. S1). The observed color change of *Ao*Cld in acetate buffer was reversible after subsequent buffer exchange to 100 mM KPi pH 7.0 (Fig. 2). The reversibility of the spectroscopic change excludes a covalent modification of the heme.

**Fig. 3:**
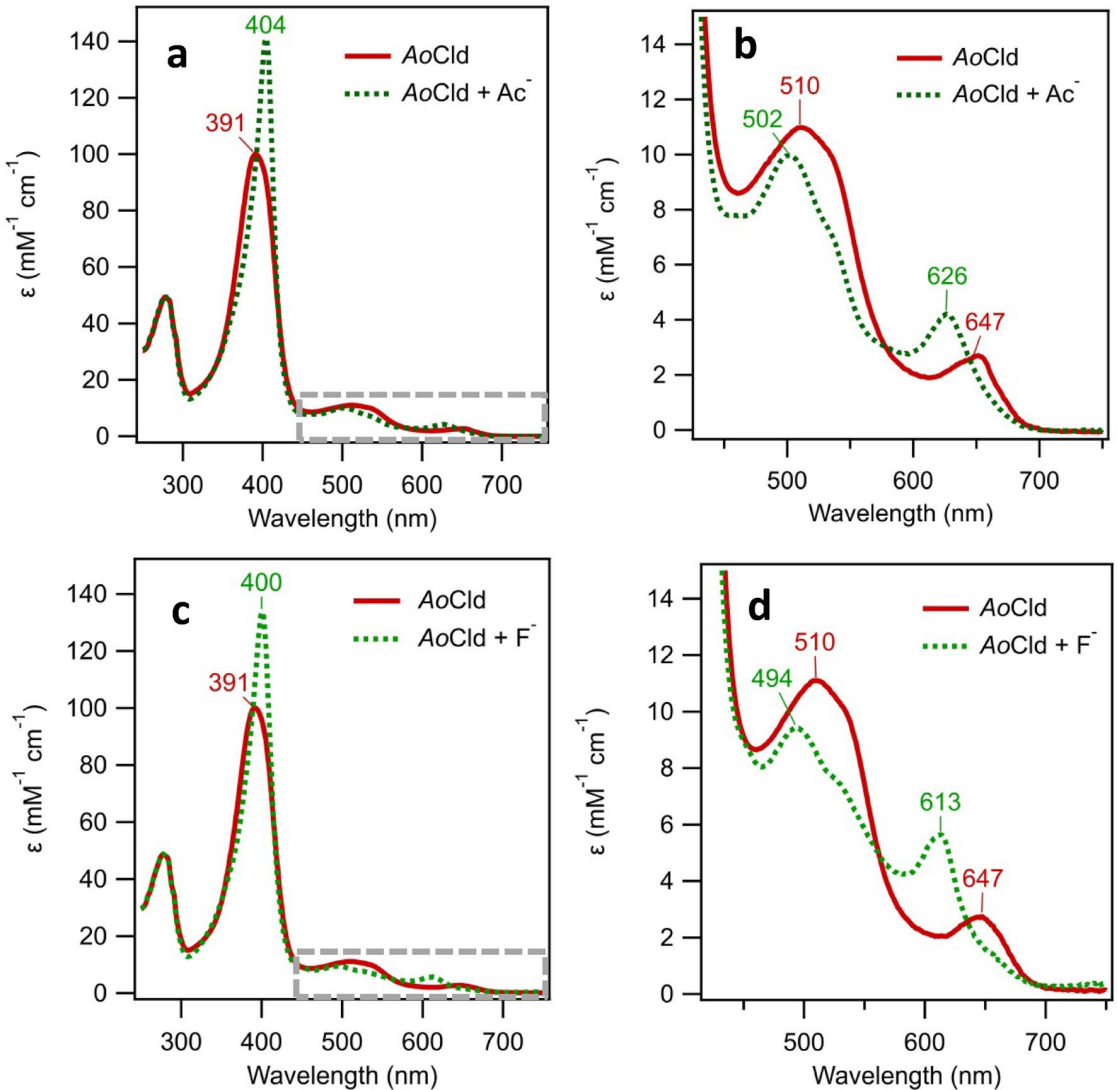
UV–visible spectra of *Ao*Cld-acetate and fluoride adducts. 10 µM *Ao*Cld solutions were recorded in 1 mL quartz cuvette with 1.0 cm path length. **a**, **b***Ao*Cld (red solid line) and *Ao*Cld-acetate adduct (green dotted line); **c**, **d***Ao*Cld (red solid line) and *Ao*Cld-fluoride adduct (green dotted line); CT bands of acetate bound *Ao*Cld. Q bands indicated by the dashed grey box in **a**, **c** are enlarged for visual clarity in **b**, **d**

The red to green color change, due to the binding of acetate can be understood by examining the UV–visible spectrum. The Soret band red-shifted from 391 nm for the five-coordinate HS ferric enzyme to 404 nm for the six-coordinate ferric acetate complex. The intensity of the Soret peak increased from 99.6 to 141 mM^−1^ cm^−1^. In the region from 450–700 nm, several changes are observed (Table 1). The CT2 and *Q* bands blue-shifted from 510 to 502 nm, and CT1 blue-shifted from 647 to 626 nm, with a concomitant twofold increase in intensity (hyperchromicity) (Fig. 3a, b). We attribute the green color to the blue-shifted CT and *Q* bands. The CT bands represent charge transfer transitions from porphyrin π a_2u_ → d_*xz*,*yz*_ (Fe), which are positioned for HS heme proteins between 450–470 nm for CT2 and 600–650 for CT1.

**Table 1:** Spectroscopic characteristics of *Ao*Cld and *Da*Cld with various ligands

Enzyme	Ligand	pH	Soret (nm)	*ε* (mM^−1^ cm^−1^)	*K*_*D*_ (μM)	References
*Ao*Cld	–	7	391	99.6	–	This work
*Da*Cld	–	7	393		–	[17]
*Ao*Cld	Ac^a^	5.8	404	141	(17.5 ± 0.5)·10^3 c^	This work
		5	404	120	(21.9 ± 3.6) ·10^3 d^	This work
*Da*Cld	Ac^a^	6.5	404		(36 ± 2) ·10^3^	[27]
*Ao*Cld	F^−^	7	400	134	(15.6 ± 1.9) ·10^3 e^	This work
*Da*Cld	F^−^	7	403		(15 ± 1) ·10^3^	[28]
*Ao*Cld	Im^b^	7	413	128	10.5 ± 2.4	This work
*Da*Cld	Im^b^	7	413		9.6 ± 0.3	[28]
*Ao*Cld	NO_2_^−^	7	412		(0.6 ± 0.2) ·10^3^	[4]
*Da*Cld	NO_2_^−^	6.5	405		(1.12 ± 0.05) ·10^3^	[27]
*Da*Cld	CN^−^	7	419		4	[28]
*Da*Cld	N_3_^−^	7	415		8.3 ± 0.1	[28]

^a^Acetate; ^b^Imidazole; ^c^average of 5 titrations, corrected for [Ac^−^] from *K*_*D*,app_ = (19.1 ± 0.5)·10^3^ mM; ^d^average of 3 titrations, corrected for [Ac^−^] from *K*_*D*,app_ = (34.4 ± 5.6) ·10^3^; ^e^average of 2 titrations

Dimeric Cld from *Klebsiella pneumonia* has been reported to bind fluoride (F^−^) resulting in a UV–visible spectrum dominated by a Soret at 404 nm and a blue-shifted CT1 band at 611 nm [15]. The F^−^ adduct of pentameric Cld from *Dechloromonas aromatica* (*Da*Cld) has similar spectral characteristics: a Soret band at 403 nm, and CT1 at 612 nm (Table 1) [10]. *Ao*Cld also binds F^−^, which is characterized by a Soret at 400 nm and a CT1 band at 613 nm (Fig. 3c, d and Table 1). The fluoride adduct of *Ao*Cld is green-colored, just like the acetate adduct.

### Direct binding of acetate and fluoride to the heme iron of *Ao*Cld

**Fig. 4:**
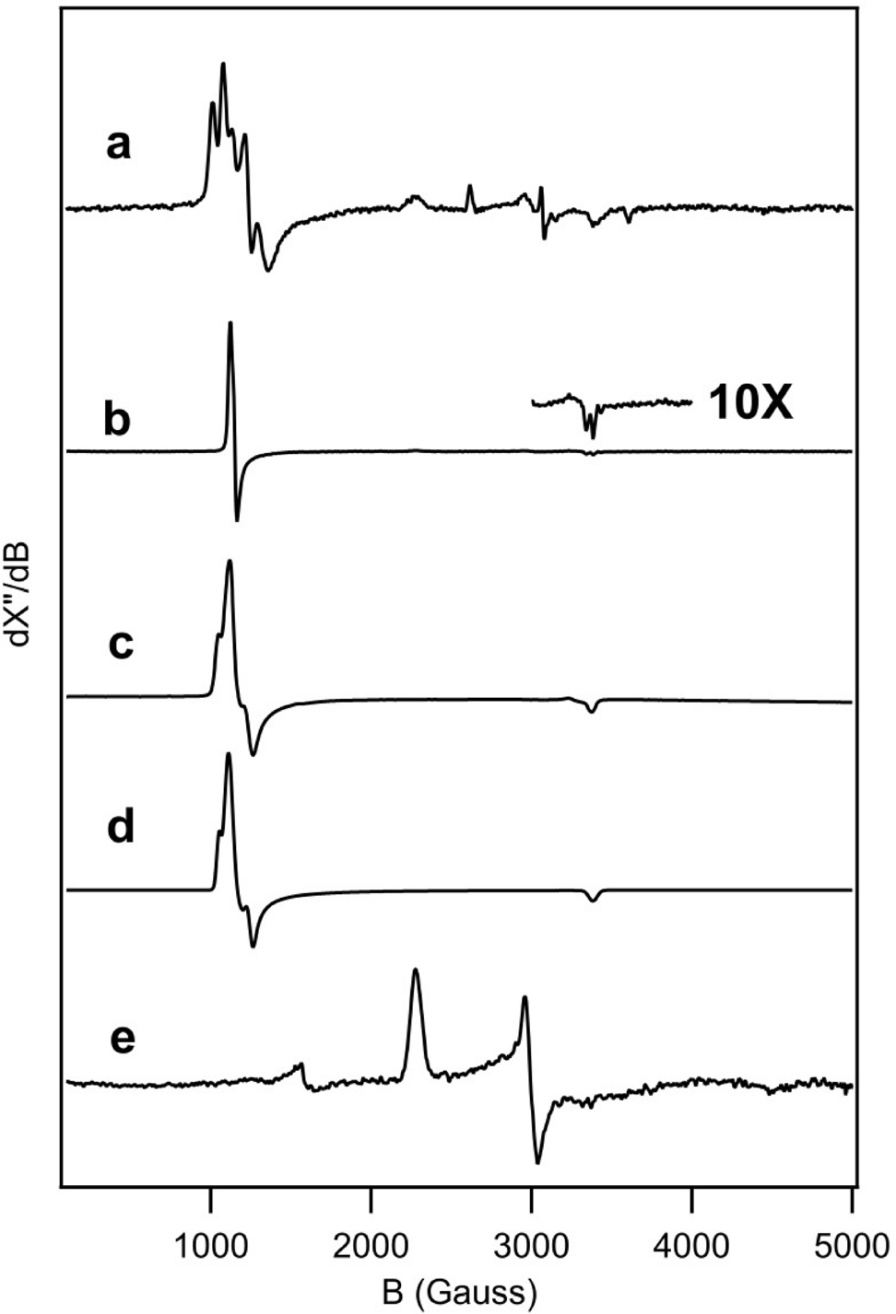
EPR spectra of *Ao*Cld without and with several ligands. **a***Ao*Cld 75 μM monomer concentration in 100 mM KPi pH 7.0. **b***Ao*Cld-fluoride adduct in 100 mM KPi pH 7.0 containing 250 mM NaF showing the ^19^F hyperfine splitting. **c***Ao*Cld-acetate adduct in 100 mM acetate pH 5.0 experimental spectrum and **d** simulation of the *Ao*Cld-acetate spectrum. Simulation parameters: two components: main component 71% *g*_*z,y, x*_ = 5.98, 5.70, 1.985; *W*_*zyx*_ = 2.7, 6.0, 3.0 mT; minor component 29% *g*_*z,y, x*_ = 6.36, 5.38, 1.985; *W*_*zyx*_ = 1.5, 2.0, 3.0 mT. **e***Ao*Cld-Imidazole adduct in 100 mM KPi pH 7.0 containing 1 mM imidazole. EPR conditions: Microwave frequency, 9.405 GHz; Microwave power, 20 mW; Modulation frequency, 100 kHz; Modulation amplitude, 1.0 mT; Temperature, 20 K. The spectra were normalized for the maximal signal amplitude

The EPR spectrum of the resting state *Ao*Cld at pH 7.0 is characterized by two HS signals (Fig. 4a and Table 2) [5]. Both signals represent *m*_*s*_ =  ± 1/2 ground state doublets of *S* = 5/2 systems, designated as ‘narrow’ and ‘broad’ according to the rhombicity *E*/*D*. The narrow signal corresponds to an *E*/*D* = 0.01–0.02 and the broad signal to an *E*/*D* = 0.03–0.04. The same signals with different ratios have been found for Cld isolated from *Azospira oryzae* strain GR-1 [4], as well as recombinantly produced *Ao*Cld, *Da*Cld and in Clds from *Ideonella dechloratans*, *Magnetospirillum* sp. [5, 7, 23].

**Table 2:** EPR parameters of *Ao*Cld and its complexes

Ligand		Spin state	*g*_*z*_	*g*_*y*_	*g*_*x*_	^a^*W*_*z*_	*W*_*y*_	*W*_*x*_	^b^*A*_*z*_	*A*_*y*_	*A*_*x*_	References
–/H_2_O	Narrow	HS	6.24	5.42	2.0	30	40	40				[4]
–/H_2_O	Broad	HS	6.70	5.02	2.0	27	50	43				
Ac^c^	Major	HS	5.98	5.70	1.99	27	60	30				This work
	Minor	HS	6.36	5.38	1.99	15	20	30				
F^−^		HS	5.90	5.90	1.995	17	17	12	20	20	44	This work
Im^d^		LS	2.96	2.25	1.51	50	50	100				[4]
H_2_O/OH^−^	High pH	LS	2.54	1.18	1.87	22	15	22				[4]
NO_2_^−^		LS	2.93	2.18	1.55	25	12	80				[4]

^a^Linewidth W in Gauss, ^b^Hyperfine coupling constant A in Gauss. ^c^Acetate; ^d^Imidazole

The EPR spectrum of the acetate adduct of *Ao*Cld is characteristic of a HS ferric species. The spectrum could be simulated assuming two components in a 71 and 29% ratio (Fig. 4c, d). The primary component is more axial than the two species in resting-state *Ao*Cld. The minor component is similar to the ‘narrow’ signal of the resting state enzyme and may represent unbound enzyme (Table 2). To our knowledge, this is the second report of the EPR spectrum of an acetate adduct of a heme enzyme.

The EPR spectrum of the fluoride adduct of *Ao*Cld shows that complex is HS ferric (Fig. 4b). The splitting of the HS signal of the fluoride adduct at *g*_*x*_ provides evidence of F^−^ binding directly to the heme iron. The splitting is due to hyperfine interaction with the ^19^F, characterized by a nuclear spin *I* = 1/2 and a hyperfine coupling constant A(^19^F) = 44 Gauss. For the myeloperoxidase fluoride adduct A(^19^F) = 35 Gauss has been reported [24]. For myoglobin and hemoglobin values of 42.9 and 44.4 Gauss have been reported, respectively [25].

Binding of the established strong field ligand imidazole resulted in a LS ferric signal characterized by *g*-values *g*_*zyx*_ = 2.96, 2.25, 1.51 (Table 2, Fig. 4e). This signal is also present as a minor component in the resting state enzyme (Fig. 4a), due to trace contamination of imidazole after the purification procedure.

The EPR shows spectral changes for the acetate and fluoride adducts indicating ligand binding to the heme iron. In the case of acetate and fluoride the heme iron remains HS, showing that these ligands are weak field ligands (Fig. 4).

### Dissociation constant of *Ao*Cld-Acetate

**Fig. 5:**
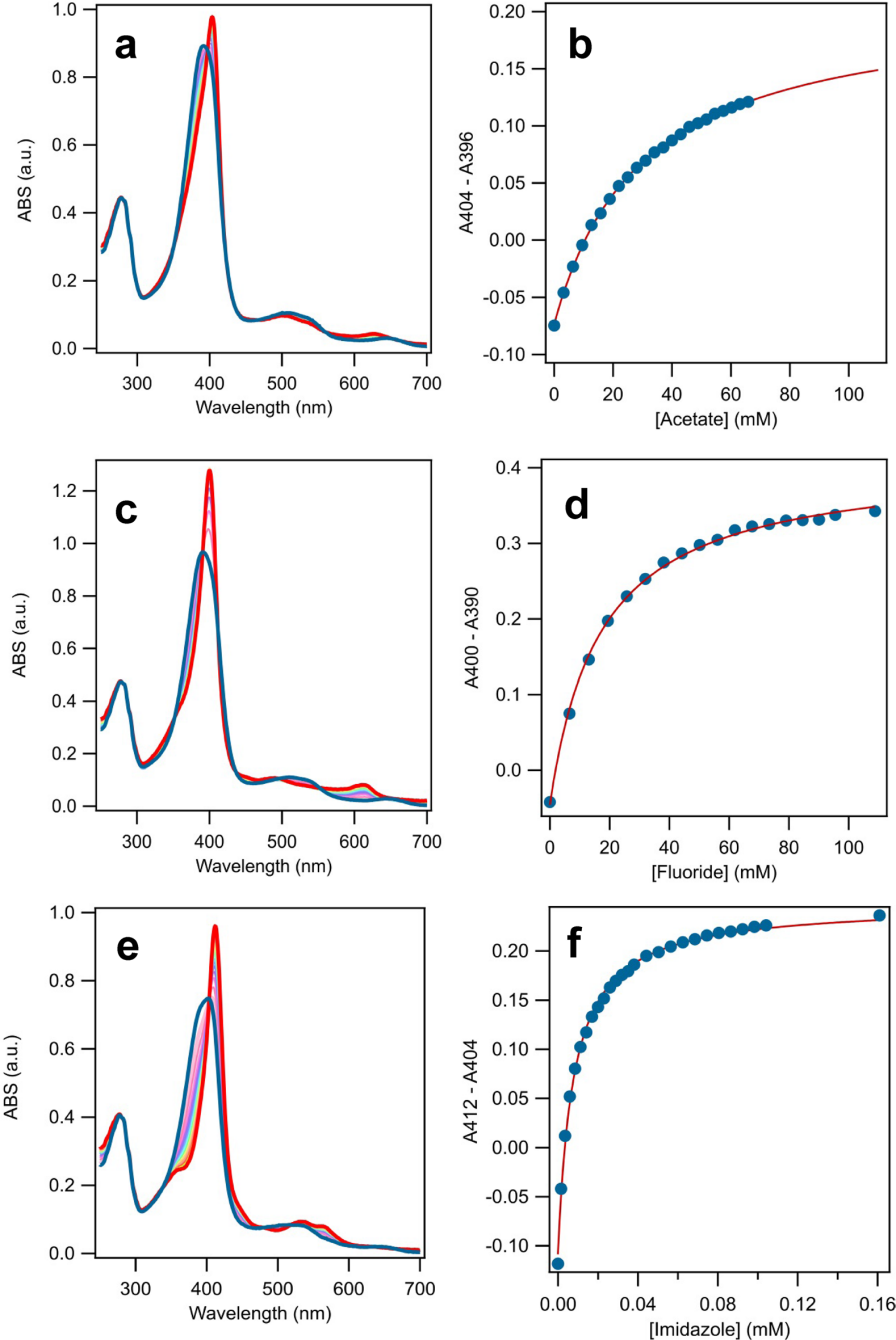
Representative equilibrium binding titrations *Ao*Cld with acetate, fluoride and imidazole. **a**,** b** titration of 8.40 μM *Ao*Cld in 100 mM citrate–phosphate pH 5.0 with 1 M sodium acetate; **c**,** d** titration of 8.97 μM *Ao*Cld in 100 mM KPi pH 7.0 with 1 M sodium fluoride; **e**,** f** titration of 8.89 μM *Ao*Cld in 100 mM Tris pH 8.0 with 2 mM imidazole. The first trace of the titration is in blue, the final trace is in red

Based on the UV–visible change of the Soret band upon ligand binding, the dissociation constants were determined under different conditions using equilibrium binding titrations (Table 1, Fig. 5 and Fig. S4–S6). The apparent dissociation constant of *Ao*Cld for acetate is *K*_*D*,app_ = 19.1 ± 0.3 mM (*n* = 3) at pH 5.8 and 34.4 ± 5.6 mM (*n* = 5) at pH 5.0. Given the *pK*_*a*_ = 4.76 of acetate the amount of ionized acetate anion, which is the most likely ligand to *Ao*Cld, is different at pH 5.0 and 5.8 [26]. Correction of the *K*_*D*,app_ values for the *pK*_*a*_ of acetate using the Henderson–Hasselbalch equation results in a *K*_*D*_ = 17.5 ± 0.5 mM at pH 5.8 and 21.9 ± 3.6 mM at pH 5.0 (calculation given in the Supplementary information). Clearly, the pH dependence of the apparent dissociation constants was consistent with the relative concentration of the ionized form of acetate. Preliminary data on acetate binding to *Da*Cld has been reported, and a *K*_*D*_ = 36 ± 2 mM was found at pH 6.5 [27].

The dissociation constant of *Ao*Cld for fluoride *K*_*D*_ = 15.6 ± 1.9 mM (*n* = 2) at pH 7.0, which is close to the reported dissociation constant for *Da*Cld (Table 1). As a reference, the binding of a well-established high-affinity ligand imidazole to *Ao*Cld was measured at different pH values (Fig. S6). The dissociation was *K*_*D*_ = 74 ± 2 μM (*n* = 1) at pH 5.0, *K*_*D*_ = 47.3 ± 5.1 μM (*n* = 3) at pH 5.8, *K*_*D*_ = 10.8 ± 2.6 μM (*n* = 3) at pH 7.0 and *K*_*D*_ = 10.5 ± 2.4 μM (*n* = 3) at pH 8.0. This is in excellent agreement with the reported *K*_*D*_ = 8.8 ± 0.2 μM at pH 7.0 for native *Ao*Cld and *K*_*D*_ = 9.6 ± 0.3 μM at pH 7.0 for native *Da*Cld (Table 1) [4, 28]. The *K*_*D*_ of Cld for imidazole is the lowest at pH 7.0 and 8.0 (Fig. S6k), which suggest that the affinity for the neutral imidazole is higher than for the imidazolium cation.

Based on the *K*_*D*_ the *ΔG’*^*0*^ for ligand binding can be calculated, which corresponds to –10.0 ± 0.06 kJ mol^−1^ for acetate (pH 5.8), − 10.3 ± 0.3 kJ mol^−1^ for fluoride (pH 7.0) and -28.3 ± 0.6 kJ mol^−1^ for imidazole (pH 7.0).

### Effect of acetate binding on *Ao*Cld activity and stability

With the exchange of buffer from acetate pH 5.0 to KPi pH 7.0, 60% specific activity was recovered after 15 min incubation in acetate buffer, pH 5.0. Acetate binding was found to affect *Ao*Cld activity. The observed *Ao*Cld specific activity in 100 mM KPi pH 7.0, 100 mM acetate buffer pH 5.0, and 100 mM citrate phosphate buffer pH 5.0 was 8.52·10^3^ U/mg, 4.96·10^3^ U/mg and 8.27·10^3^ U/mg, respectively. It was also observed that acetate buffer pH 5.0 affected the enzyme stability. Prolonged incubation (2 h) resulted in a 90% activity loss (Fig. S3).

## Discussion

### Acetate binds directly to the heme iron in *Ao*Cld

Acetic acid has a *pK*_*a*_ = 4.76 [26]. At pH 5.8 approximately 93% of the acid is present in its ionized form as calculated using HySS [29]. We conclude that acetate is an anionic ligand for *Ao*Cld. The blue-shift of the CT1 band in the UV–visible spectrum, similar to the binding of fluoride, is evidence of acetate binding directly to the heme iron. Furthermore, the EPR spectrum of the acetate adduct showed a novel HS signal that is significantly more axial than the HS signals observed for the unliganded enzyme. The binding of acetate as a direct ligand to Fe is rare and has been observed, to our knowledge, only for one other heme protein: leghemoglobin. Leghemoglobin is a high-affinity oxygen-binding protein, related to myoglobin, produced by legume plants in root nodules as part of the symbiotic relationship with nitrogen-fixing bacteria. Wittenberg et al. showed that acetate strongly reduced the rate of ferric iron reduction by sodium dithionite [30]. The UV–visible and EPR spectral properties of the acetate complex provided evidence for a high-spin complex associated with a subtle blueshift of the CT1 band from 627 to 622 nm [31]. Leghemoglobin has a distal histidine that forms a hydrogen bond with oxygen in oxyleghemoglobin [32]. It is likely that the distal histidine is also involved in binding of acetate to this protein in the ferric state.

Acetate binding to a distal site in HRP has been reported [33, 34]. The *K*_*D*_ of HRP for acetate is > 300 mM. A small redshift of the CT1 band was observed. The Soret band did not change, but an increased asymmetry of the peak was observed. EPR spectroscopy of the HRP-acetate adduct showed the complex is HS with g-values that are similar to the unliganded enzyme. The structure of HRP-acetate showed that acetate was bound parallel to the heme plane with both carboxylate oxygens H-bonding to the proximal Arg [34]. The minimal distance between the oxygen atoms of acetate and the iron was 3.6 Å, which showed that there is no direct coordination of acetate to the iron in HRP.

### Why is the *Ao*Cld-acetate complex green?

Both the Soret and Q bands represent π → π* transitions of the porphyrin ring system. The charge transfer bands from 500–600 nm represent *π* a_2u_ → d_*xz*,*yz*_ (Fe) based on a study with myoglobin single crystals [35]. *Ao*Cld in KPi pH 7.0 has the characteristic UV–visible spectrum of a 5-coordinate HS ferric heme enzyme, with a broad Soret band at 391 nm, broad overlapping Q bands at 510 nm and a clear CT1 band at 647 nm. The green color of *Ao*Cld in acetate buffer is caused by the blueshift of the CT1 band due to direct binding of acetate to the heme iron in the enzyme, in a similar way as for fluoride which also forms a green-colored complex with *Ao*Cld. We conclude that for any weak field ligand binding to HS ferric Cld, which is anionic or at least a good Lewis base a similar spectral change is expected and hence the resulting complex will also have a green color.

The interaction of the *p*-orbitals of the ligand with the *d*-orbitals of the heme iron causes an increase in the energy of the metal *d*-orbitals, which is responsible for the blueshift of the CT1 band. The stronger this ligand to metal interaction, the stronger the blueshift. Hydrogen bonding of a distal amino acid residue with the ligand influences the strength of the interaction. A hydrogen bond donor would lead to a stronger interaction between the ligand and the metal, leading to a stronger blueshift of the CT1 band. A hydrogen bond acceptor would lead to a redshift of the CT1 band. The CT1 band of the fluoride adduct has been considered to be a sensitive probe for the strength of the H-bonding donation of the distal amino acid residues to the F^−^ ion. The CT1 maximum has been considered to be sensitive to the distal ligand interaction [36]. The CT1 band is sensitive to the polarity of the axial ligand as well. For example, a blueshift of 8 nm has been observed for an exchange of the axial His to a Glu, i.e. exchanging for a more negative ligand, in the 5-coordinate HS ferric cytochrome c peroxidase [37].

### *Ao*Cld-acetate complex in comparison to other green heme enzymes

Spectroscopic changes that give heme proteins a green color have been reported with different causes. Oxidation of heme to verdoheme catalyzed by heme oxygenase as part of the oxidative heme degradation pathway results in such a color change [38]. The UV–visible spectrum of verdoheme is characterized by a Soret band with a reduced intensity at 405 nm and a broad absorbance band from 650–700 nm. Additionally, naturally occurring green heme proteins have been reported, the most prominent one being myeloperoxidase. It is interesting that myeloperoxidase catalyzes the formation of hypochlorite from chloride and hydrogen peroxide, which is in some ways reminiscent of the reversed reaction of chlorite dismutase. The UV–visible spectrum of myeloperoxidase is characterized by a Soret band at 428 nm and α band at 570 nm, which has been attributed to a red-shifted α band [39]. The characteristic spectral properties of myeloperoxidase are due to three covalent heme-protein bonds, which cause considerable bending of the heme plane leading to the out-of-plane location of the iron [40]. Other naturally occurring green heme proteins have also been isolated from microorganisms such as the *Neurospora crassa* catalase and the green heme protein from the purple acid bacterium *Halochromatium salexigens* [41, 42]. Finally, the seminal intermediate in many heme enzymes, compound I, an oxoferryl porphyrin π cation radical, is green-colored, e.g. in Cytochrome P450 and HRP [43, 44].

### Why is the binding of acetate to *Ao*Cld similar to F^−^ binding and not Cl^−^ binding?

The acetate complex of Cld could offer an interesting structural model for the Michaelis complex of the enzyme. In chlorite, the O–Cl–O bond angle is 111°, whereas the O–C–O bond angle in acetate is 129° [45]. The Cl–O bond length in chlorite is 156 pm, whereas the C–O bond length in acetate is 127 pm. The required hydrogen bonding interaction between bound acetate and Arg183 could be similar to the Michaelis complex structure.

The higher affinity of Cld for F^−^ as compared to Cl^−^ is related to the Lewis base hardness, F^−^ being a harder acid than Cl^−^ and binding better to the borderline hard acid Fe^3+^. F^−^ is a weak field ligand in the context of ligand field theory, as it is a π donor ligand leading to a small Δ_o_. The spin state data in Table 2 are consistent with the spectrochemical series. Acetate as a ligand in inorganic chemistry has been reported to be close to the water in the spectrochemical series [46, 47]. This is consistent with the HS state iron in the Cld-Acetate adduct.

The blueshift and increase of intensity of the CT1 band for the acetate and fluoride complexes are related to the *π* donor character of both ligands. The acetate and fluoride complexes are likely stabilized by hydrogen bonding interaction with Arg183. Mutagenesis of Arg183 to Ala results in the complete loss of affinity for fluoride and acetate, but not for imidazole (Fig. S7).

Interestingly Cl^−^ is not a suitable ligand for Cld, even though it is a product of the reaction. Cl^−^ is a much weaker and softer base as compared to F^−^. Cl^−^ binding to Cld has been reported before, but for *Da*Cld no affinity could be measured, for the dimeric *Klebsiella pneumoniae* Cld cooperative binding with a *K*_*D*_ of 1.4 mM and a Hill coefficient of 2.3 was found [15], and only a very weak affinity was observed for *Ao*Cld as no saturation was observed up to 2.5 M (not shown).

The distal Arg is important for high-affinity binding of F^−^, CN^−^ and N_3_^−^, but not imidazole in *Da*Cld [16]. This strengthens the idea that the binding of the anionic ligands involves hydrogen bonding with the distal Arg in a so-called closed conformation. We have shown that acetate binding also likely involves hydrogen bonding to Arg183.

### Physiological relevance of the binding of acetate to *Ao*Cld

There are not many reports on the absolute intracellular acetate concentration in bacteria. The original organism from which this Cld was isolated, *Azospira oryzae* GR-1, grows anaerobically on a mineral medium with 2 g L^−1^ (24 mM) sodium acetate as sole carbon and electron source and perchlorate as electron acceptor [48]. The expression level of chlorite dismutase in *A. oryzae* GR-1 under these conditions has been estimated to be high, circa 7% of the total protein. Native chlorite dismutase has a leader sequence for periplasmic targeting. It is not unlikely that the periplasmic acetate concentration is similar to the extracellular acetate concentration and, therefore, circa 50% of natively expressed Cld may be acetate bound. The *K*_*i*_ and mechanism of inhibition by acetate was not determined, so we cannot estimate the precise effect on the activity in the presence of a certain local chlorite concentration. However, in conclusion, it is possible that the acetate effect we report has physiological relevance and may offer a link between the central carbon metabolism and the anaerobic respiratory pathway in this organism.

Here we present that *Ao*Cld binds acetate directly to iron in the ferric state. The resulting high spin ferric complex is characterized by a marked blueshift and hyperchromicity of the CT1 band, which gives the complex a green color. Similar effects can be expected for any ligand binding directly to the heme iron forming a high-spin ferric complex, as is indeed the case for fluoride.

## Electronic supplementary material

Below is the link to the electronic supplementary material.

Supplementary file1 (PDF 602 kb)

## Abbreviations

Ac: Acetate
*Ao*Cld: *Azospira oryzae* Chlorite dismutase
Cld: Chlorite dismutase
*Da*Cld: *Dechloromonas aromatica* Chlorite dismutase
EPR: Electron paramagnetic resonance
HS: High spin
HRP: Horseradish peroxidase
Im: Imidazole
IMAC: Immobilized metal ion affinity chromatography
IPTG: Isopropyl ß-d-1-thiogalactopyranoside
KPi: Potassium phosphate buffer
LS: Low spin
Rz: Reinheitszahl
