# Supplementary material for: A traffic light enzyme: acetate binding reversibly switches chlorite dismutase from a red- to a green-colored heme protein
Source: J Biol Inorg Chem. 2020 Apr 3;25(4):609–20. doi: 10.1007/s00775-020-01784-1 (PMC7239840; doi:10.1007/s00775-020-01784-1)
Supplement: Supplementary file 1 — Supplementary file1 (PDF 602 kb) [file 775_2020_1784_MOESM1_ESM.pdf]

Electronic Supplemenatary Material

A traffic light enzyme: acetate binding reversibly switches chlorite dismutase from a red- to a green-colored heme protein

Durga Mahor[1], *Julia Püschmann*[1], *Menno van den Haak*[1], *Pepijn J. Kooij*[1], *David L.J. van den Ouden*[1], *Marc J.F. Strampraad*[1], *Batoul Srour*[1,2], *Peter-Leon Hagedoorn*[1]*

[1]Department of Biotechnology, Delft University of Technology, Van der Maasweg 9, 2629HZ, Delft, The Netherlands

[2]Present address: Institute for Integrative Biology of the Cell (I2BC), CEA, CNRS, Univ. Paris-Sud, Université Paris-Saclay, 91198 Gif-sur-Yvette cedex, France.

*Corresponding author

e-mail: p.l.hagedoorn@tudelft.nl

phone: +31-(0)15-2782334

ORCID P.L. Hagedoorn: 0000-0001-6342-2022

Contents

Fig. S1 The color of *Ao*Cld in different buffers 1

Table S1 Bacterial strains and plasmid used in this work 2

Fig. S2 Purification *of Ao*Cld 3

Table S2 Purification table for *Ao*Cld produced in *E. coli* 3

Fig. S3 Stability of *Ao*Cld in acetate buffer pH 5.0 4

Fig. S4 Equilibrium binding titrations of acetate to *Ao*Cld 5

Correction of K_D for acetate binding to *Ao*Cld for the K_D of acetate 6

Fig. S5 Equilibrium binding titrations of fluoride to *Ao*Cld 7

Fig. S6 Equilibrium binding titrations of imidazole to *Ao*Cld 8

Fig. S7 Ligand binding to R183A *Ao*Cld 10

Fig. S1 The color of *Ao*Cld in different buffers

250 µM of *Ao*Cld was transferred into 100 mM of 1 mL volume of respective buffers.

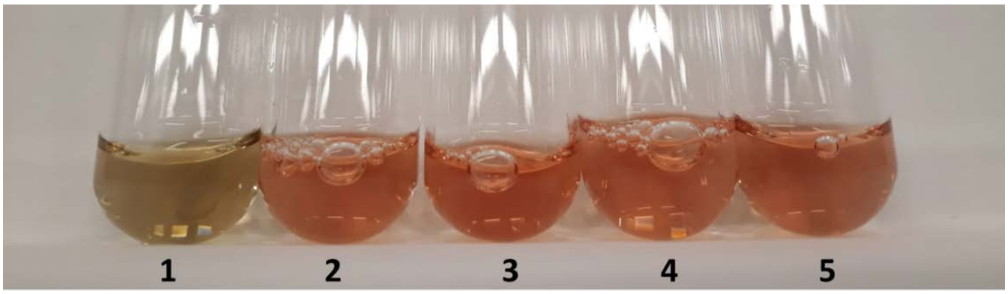

1 Acetate buffer pH 5
2 Citrate (phosphate) buffer pH 5
3 Citrate (sodium) buffer pH 5
4 Phosphate buffer pH 7
5 Tris buffer pH 8

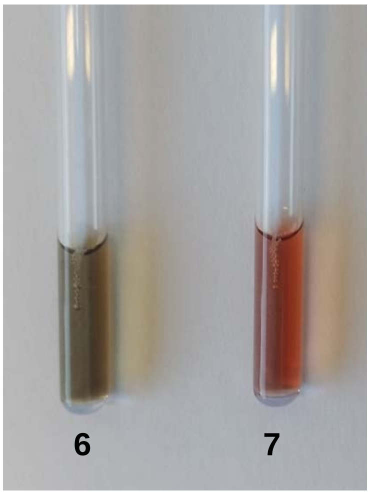

6 Acetate buffer pH 5
7 Citrate phosphate buffer pH 5.8

Table S1 Bacterial strains and plasmid used in this work

	**Phenotype or characteristics**	**Source or reference**
Strains		
E. coli TOP10	*mcrA, Δ(mrr-hsdRMS-mcrBC), φ80lacZ(del)M15, ΔlacX74, deoR, recA1, araD139, Δ(ara-leu)7697, galU, galK, rpsL(SmR), endA1, nupG*	Invitrogen
E. coli BL21 (DE3) pLysS	F^{-}, *ompT*, $hsdS_B$ (r_B–, m_B–), *dcm, gal, λ(DE3), pLysS*, Cm^r	Invitrogen
Plasmids		
pET28-*Ao*Cld	Expression *A. oryzae* Cld with his-tag	De Geus et al. [1]

[1] D. C. de Geus, E. A. J. Thomassen, C. L. van der Feltz and J. P. Abrahams (2008) Acta Cryst F 64:730-732

Fig. S2 Purification *of Ao*Cld

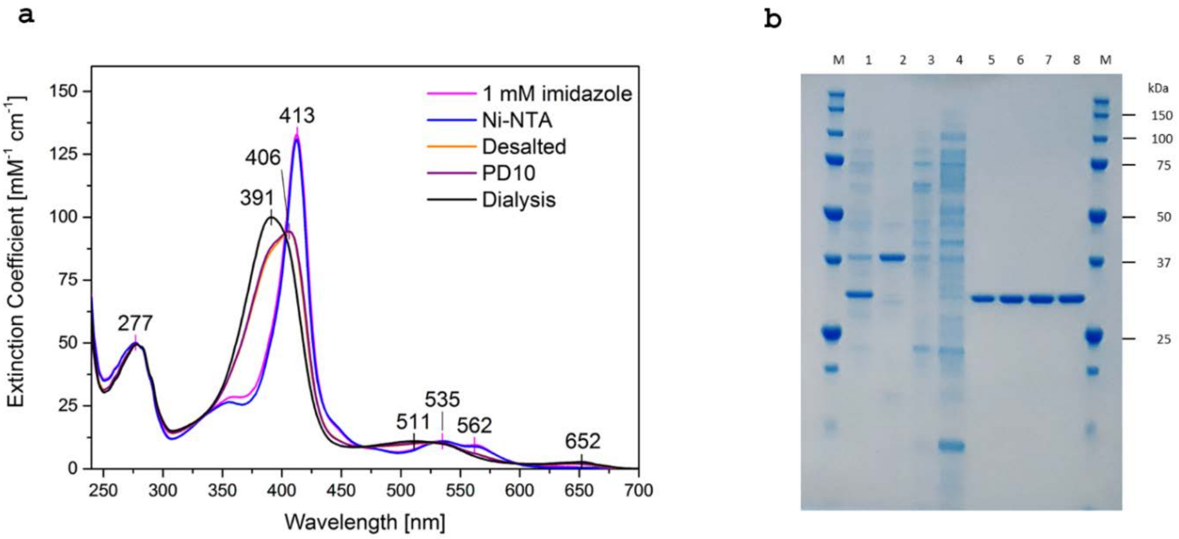

a UV-visible spectrum of *Ao*Cld at different protein purifications steps: 10 µM *Ao*Cld in 100 mM KPi pH 7.0 at 21 °C (black line), *Ao*Cld with1 mM imidazole (pink line), *Ao*Cld after Ni-NTA step (blue line), purified *Ao*Cld after HiTrap desalting step (orange line), purified *Ao*Cld after PD10 desalting step (purple line); **b** SDS-PAGE profile of purified *Ao*Cld: 10 µg *Ao*Cld was loaded in each well of 12% SDS-PAGE. M: Precision Plus Protein Blue Standard Marker from Bio-Rad, 1: Cell lysate, 2: Cell pellet, 3: HisTrap flow through, 4: HisTrap last wash step, 5: HisTrap purified protein, 6: HiTrap desalted protein, 7: PD10 desalted protein, 8: Dialyzed protein

Table S2 Purification table for *Ao*Cld produced in *E. coli*

**Purification step**	**Specific activity (10^3 U/mg)**	**Yield (%)**	**Purification fold**	**Rz ratio (A_{391nm}/A_{278nm})**
Cell Lysis	2.86 ± 0.18	100	1.0	-
Affinity Chromatography (Ni-Sepharose)	8.84 ± 0.14	71	3.1	1.05
Desalting (HiTrap)	9.54 ± 0.35	58	3.3	1.76
Desalting (PD10)	9.82 ± 0.45	49	3.4	1.80
Dialysis	8.52 ± 0.32	40	3.0	2.04

Fig. S3 Stability of *Ao*Cld in acetate buffer pH 5.0

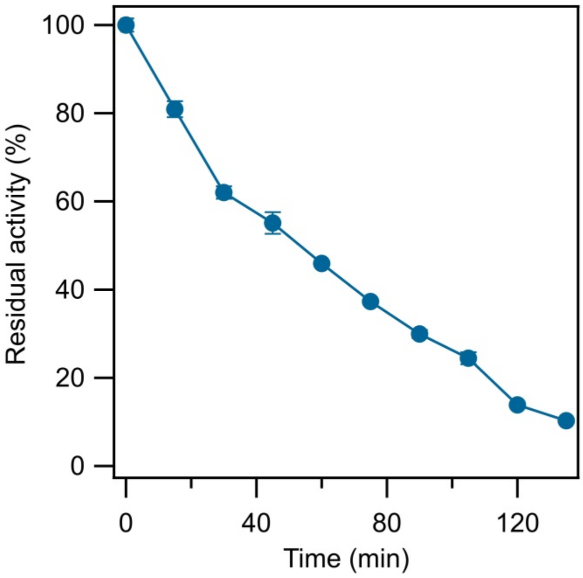

*Ao*Cld enzyme solution incubated in 100 mM acetate buffer pH 5.0 for different time periods then activity was measured with the Clark electrode using 1 mM sodium chlorite, 120 pM pre-incubated enzyme in 100 mM KPi pH 7.0 at 20 °C. Experiments were performed in triplicate.

Fig. S4 Equilibrium binding titrations of acetate to *Ao*Cld

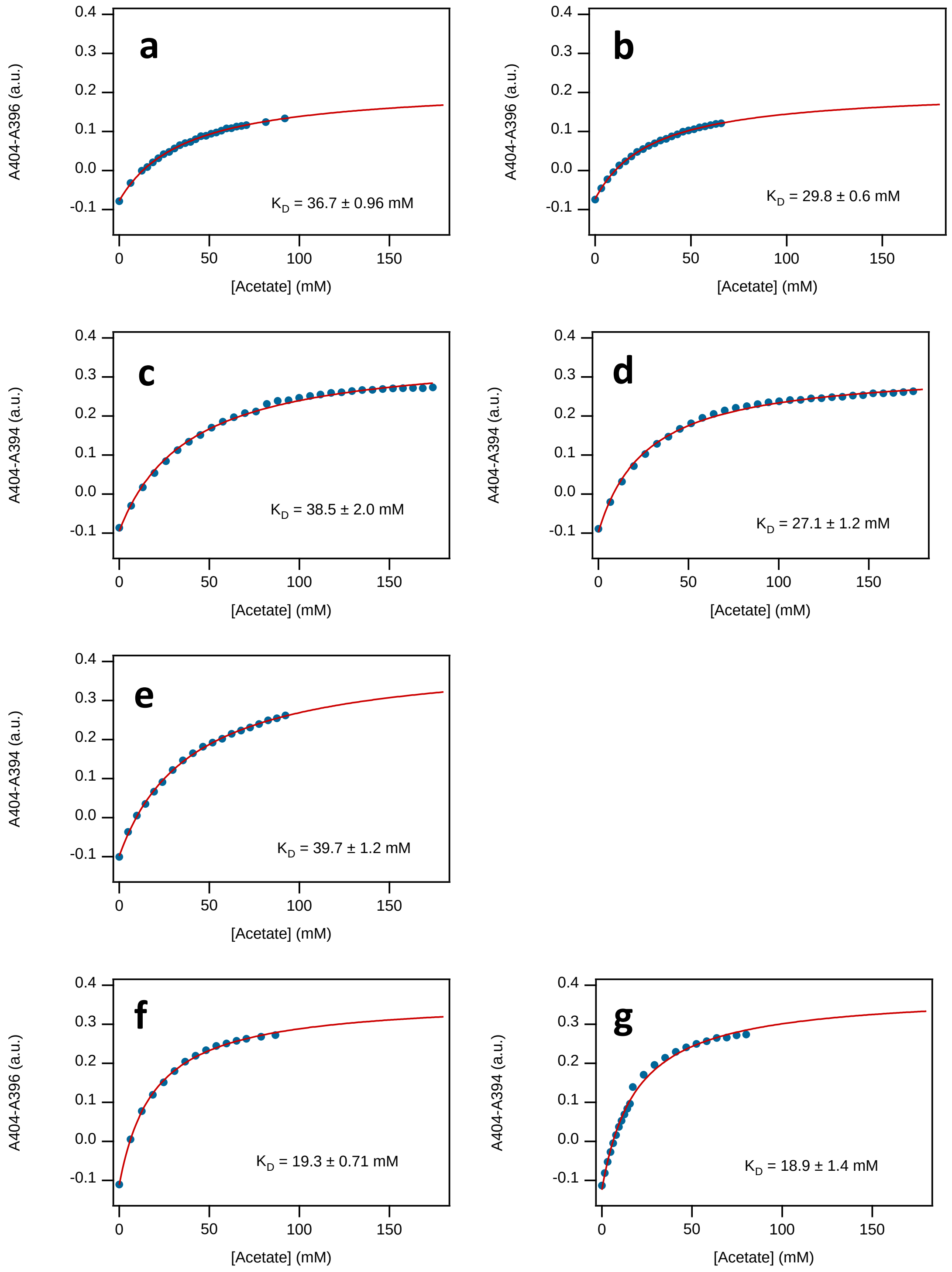

a Titration of 8.61 µM *Ao*Cld in 100 mM citrate-phosphate pH 5.0 with 1 M sodium acetate, **b** Titration of 8.40 µM *Ao*Cld in 100 mM citrate-phosphate pH 5.0 with 1 M sodium acetate, **c** Titration of 9.83 µM *Ao*Cld in 100 mM citrate-phosphate pH 5.0 with 2 M sodium acetate, **d** Titration of 9.73 µM *Ao*Cld in 100 mM citrate-phosphate pH 5.0 with 2 M sodium acetate, **e** Titration of 10.5 µM *Ao*Cld in 100 mM citrate-phosphate pH 5.0 with 0.75 M sodium acetate, **f** Titration of 9.04 µM *Ao*Cld in 100 mM KPi pH 5.8 with 1 M sodium acetate, **g** Titration of 9.23 µM *Ao*Cld in 100 mM KPi pH 5.8 with 1 M sodium acetate

Correction of K_D for acetate binding to *Ao*Cld for the K_D of acetate

Acetate has a pK_a = 4.756.[2] For acetate the ionization equilibrium is as follows:

$$\underset{Ac^-}{H_2COO^-} + H^+ \rightleftharpoons \underset{HAc}{H_2COOH}$$

According to the Henderson-Hasselbalch equation we obtain the following:

$$pH = pK_a + log\left(\frac{[Ac^-]}{[HAc]}\right)$$

At pH 5.0: $\frac{[Ac^-]}{[HAc]} = 1.75$

At pH 5.8: $\frac{[Ac^-]}{[HAc]} = 11.1$

The apparent K_D was obtained using the total acetate concentration $[Ac_{total}] = [Ac^-]+[HAc]$.

At pH 5.0: $[Ac_{total}] = [Ac^-] + [HAc] = \left(1 + \frac{1}{1.75}\right)[Ac^-] = 1.57[Ac^-]$

At pH 5.8: $[Ac_{total}] = [Ac^-] + [HAc] = \left(1 + \frac{1}{11.1}\right)[Ac^-] = 1.09[Ac^-]$

We can now correct the apparent dissociation constant by correcting:

$$K_{D,app} = \frac{[Ac_{total}][Cld]}{[Ac-Cld]} = \frac{1.57[Ac^-][Cld]}{[Ac-Cld]} = 1.57 \cdot K_D$$

At pH 5.0: $K_{D,app} = 1.57K_D$ and so $K_D = 0.64K_{D,app} = 0.64 \cdot 34.4 = 21.9$ mM

At pH 5.8: $K_{D,app} = 1.09K_D$ and so $K_D = 0.92K_{D,app} = 0.92 \cdot 19.1 = 17.5$ mM

[2] W. M. Haynes, D. R. Lide and T. J. Bruno (2016) CRC handbook of chemistry and physics: A ready-reference book of chemical and physical data. CRC Press, Boca Raton, FL, USA

Fig. S5 Equilibrium binding titrations of fluoride to *Ao*Cld

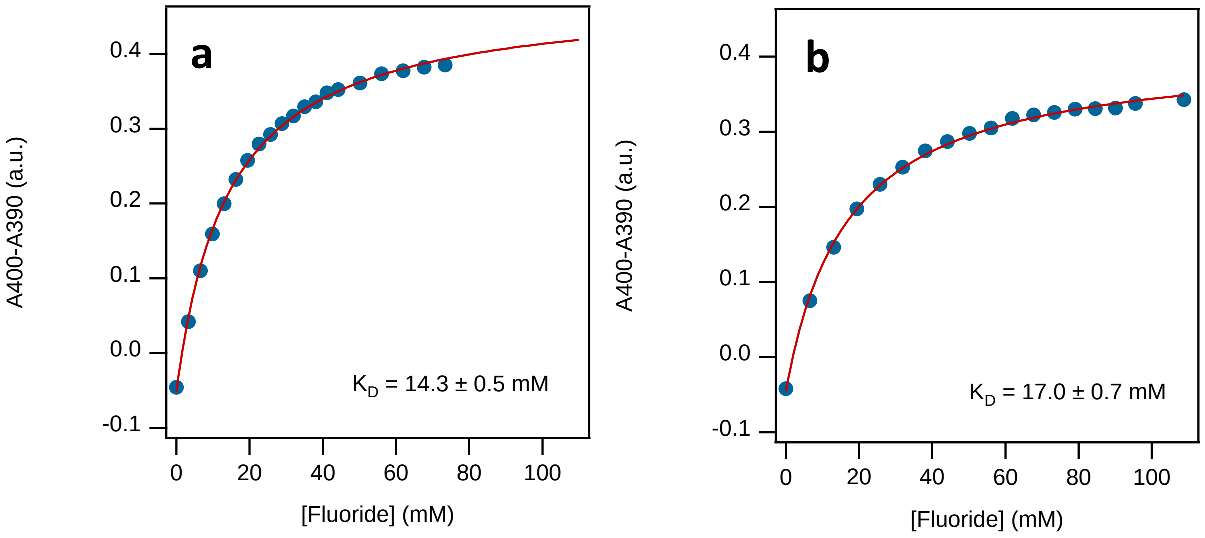

a Titration of 9.92 µM *Ao*Cld in 100 mM KPi pH 7.0 with 1 M sodium fluoride, **b** Titration of 8.97 µM *Ao*Cld in 100 mM KPi pH 7.0 with 1 M sodium fluoride

Fig. S6 Equilibrium binding titrations of imidazole to *Ao*Cld

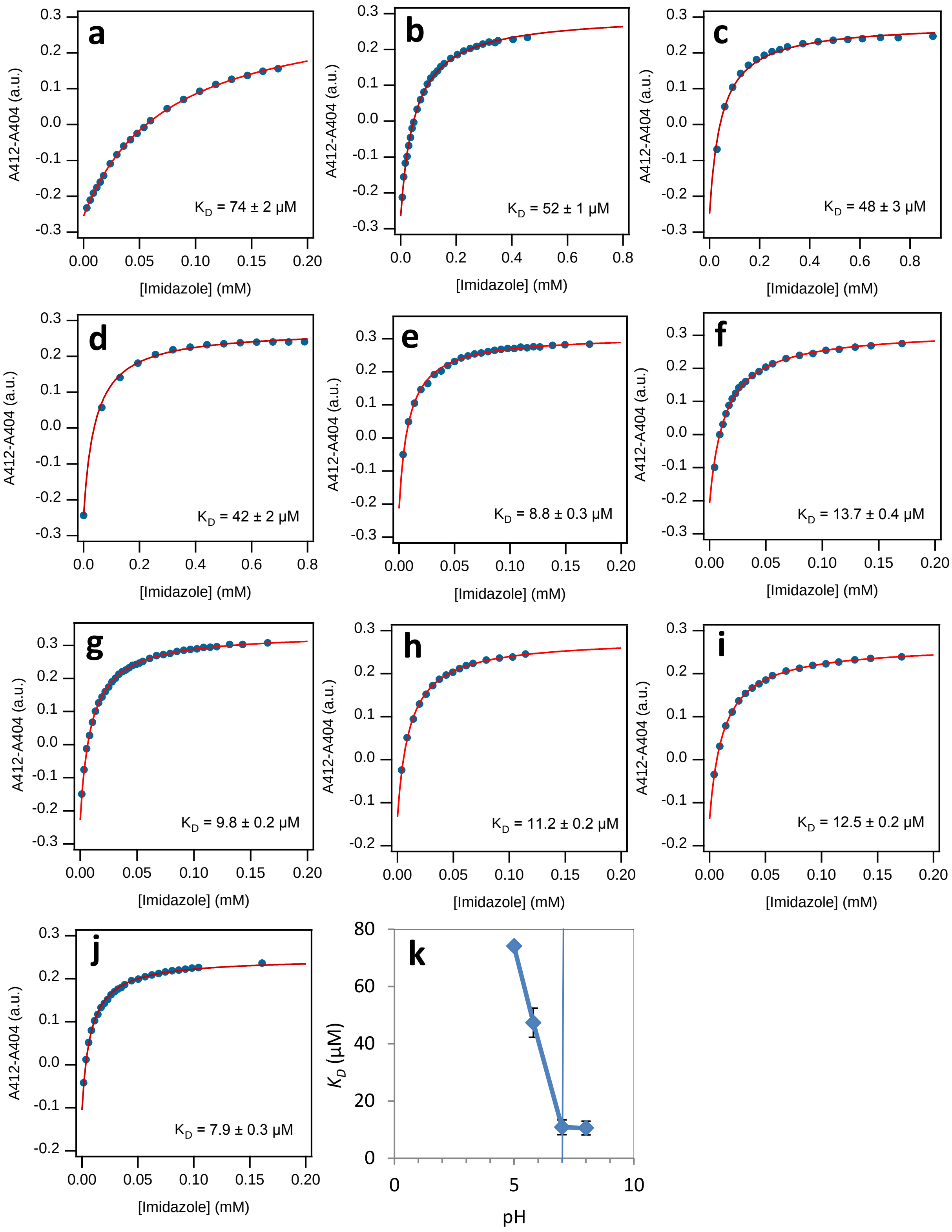

a Titration of 9.50 µM *Ao*Cld in 100 mM KPi pH 5.0 with 2 mM imidazole **b** Titration of 10.3 µM *Ao*Cld in 100 mM KPi pH 5.8 with 4 mM imidazole, **c** Titration of 8.80 µM *Ao*Cld in 100 mM KPi pH 5.8 with 10 mM imidazole, **d** Titration of 8.68 µM *Ao*Cld in 100 mM KPi pH 5.8 with 10 mM imidazole, **e** Titration of 9.50 µM *Ao*Cld in 100 mM KPi pH 7.0 with 2 mM imidazole, **f** Titration of 10.6 µM *Ao*Cld in 100 mM KPi pH 7.0 with 2 mM

imidazole, **g** Titration of 9.88 µM *Ao*Cld in 100 mM KPi pH 7.0 with 2 mM imidazole, **h** Titration of 10.2 µM *Ao*Cld in 100 mM Tris pH 8.0 with 2 mM imidazole, **i** Titration of 9.60 µM *Ao*Cld in 100 mM Tris pH 8.0 with 2 mM imidazole, **j** Titration of 8.89 µM *Ao*Cld in 100 mM Tris pH 8.0 with 2 mM imidazole, **k** pH dependence of K_D of *Ao*Cld – imidazole complex. The straight blue line indicates the pK_a = 7.0 of imidazolium-imidazole equilibrium.[3]

[3] H. Walba and R. W. Isensee (1961) J Org Chem 26:2789-2791

Fig. S7 Ligand binding to R183A *Ao*Cld

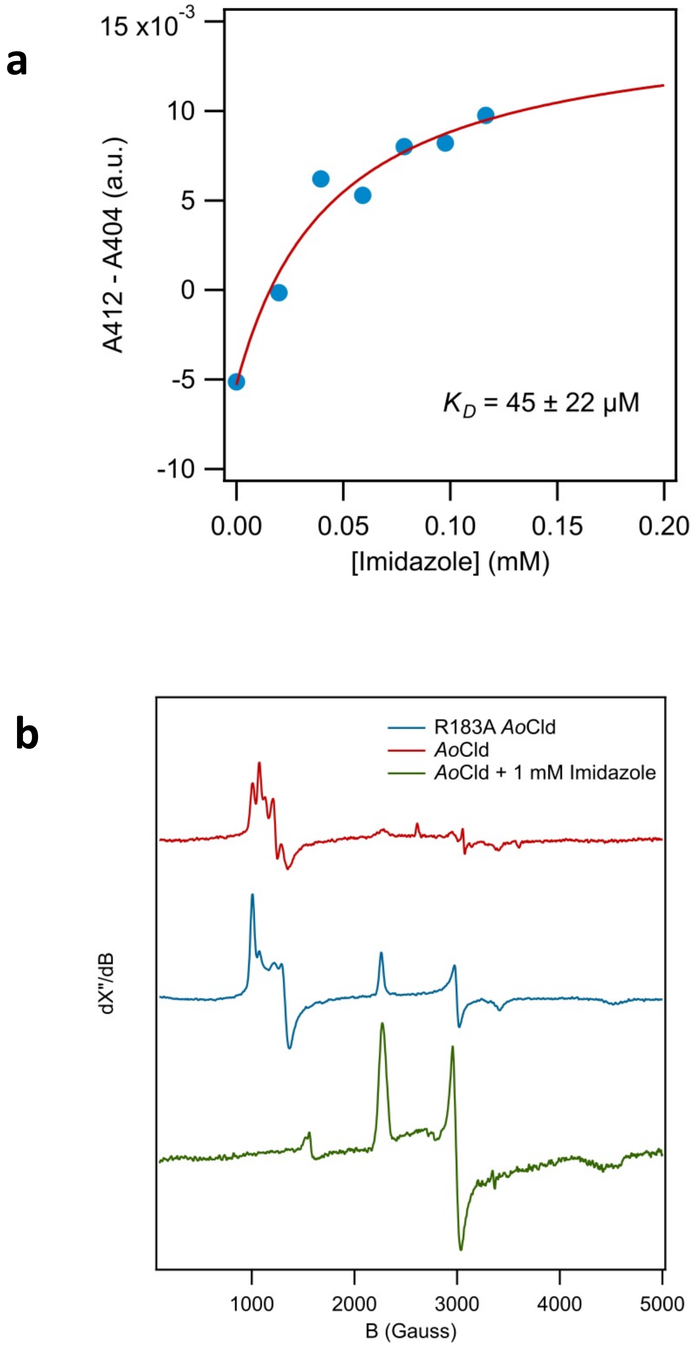

a Titration of 0.3 µM *Ao*Cld in 100 mM Tris pH 8.0 with 2 mM imidazole, **b** EPR spectroscopy of 75 µM *Ao*Cld (red line) in 100 mM KPi pH 7.0, 75 µM R183A *Ao*Cld (blue line) in 100 mM KPi pH 7.0 and 75 µM *Ao*Cld with 1 mM imidazole (green line) in 100 mM KPi pH 7.0. EPR conditions: Microwave frequency, 9.405 GHz; Microwave power, 20 mW; Modulation frequency, 100 kHz; Modulation amplitude, 1.0 mT; Temperature, 20 K.
